# *Ex Vivo* Differential Responsiveness to *Clostridium perfringens* and *Lactococcus lactis* by Avian Small Intestine Macrophages and T Cells

**DOI:** 10.3389/fimmu.2022.807343

**Published:** 2022-02-09

**Authors:** Nitish Boodhoo, Bahram Shojadoost, Mohammadali Alizadeh, Raveendra R. Kulkarni, Shayan Sharif

**Affiliations:** ^1^ Department of Pathobiology, Ontario Veterinary College, University of Guelph, Guelph, ON, Canada; ^2^ Department of Population Health and Pathobiology, College of Veterinary Medicine, North Carolina State University, Raleigh, NC, United States

**Keywords:** *Clostridium perfringens*, avian intestinal immunity, *Lactococcus lactis*, T cell, macrophages, probiotic

## Abstract

Tissue resident immune system cells in the chicken intestine play a significant role in the protection against pathogens. However, very little is known about these cells. The current study was conducted to further characterize chicken intestinal immune system cells. Furthermore, this study aimed to assess the immune modulatory action of a highly virulent *Clostridium perfringens*, a commonly found chicken intestinal microbe, in comparison with a non-commensal, *Lactococcus lactis*, on intestine-derived immune system cells. The results demonstrated varying distribution of innate and adaptive immune cells along the avian gut-associated lymphoid tissue (GALT) in the duodenum, jejunum, ileum, and cecal tonsils. In addition, steady-state and tissue-specific presence of CD25+ cells among αβ and γδ T-cell subsets was assessed along the intestine. *Ex vivo* stimulation with *C. perfringens* or *L. lactis* resulted in a significant increase in the frequency of CD25+ T cells (γδ and αβ T cells). In addition, significantly more cell death was observed in *ex vivo* stimulation with *C. perfringens*, which was indirectly correlated with a decrease in macrophage activation based on nitric oxide (NO) production with no effect on lymphoid cell responsiveness as per intracellular interferon (IFN)-gamma (γ) staining. *Ex vivo* stimulation with *L. lactis* activated γδ T cells and αβ T cells, based on intracellular IFN-γ staining, while it had limited effect on macrophages. However, the ability of γδ and αβ T cells to produce IFN-γ and the ability of macrophages production of NO was rescued in the presence of *L. lactis.* These results demonstrate the potential application of *L. lactis*, as a probiotic, against virulent *C. perfringens* infection in chicken.

## Introduction

*Clostridium perfringens*, since its first descriptions by Parish (1961) in poultry (1), continues to be a persisting problem for the poultry industry (2). In the growing broiler chicken, both avirulent and highly virulent *C. perfringens* strains effectively colonize the intestine without causing a disease (3). However, under certain circumstances, colonization by highly virulent strains leads to necrotic enteritis. Infection with an opportunistic and highly virulent *C. perfringens* expressing an array of toxins (α-toxin, necrotic enteritis B-like toxin; NetB and TpeL), in the presence of *Eimeria*, is a requirement for mucosal perturbation, loss of intestinal epithelial integrity, and subsequent translocation of bacteria through the mucus layer into deeper tissues (4). Furthermore, necrotic enteritis (NE) pathogenesis can be exacerbated by high protein diet, wheat and barley diet, or immunosuppression as a result of viral infections (5). Even as maternal antibody levels dissipate, the intestinal mucosal barrier and gut-associated lymphoid tissue (GALT) can protect the growing chickens against *C. perfringens*-induced NE (6). However, intestine colonizing NetB and TpeL-negative *C. perfringens* strains are not sufficient to elicit a protective immune response against highly virulent *C. perfringens* infection and subsequent progression of NE (3, 7). Therefore, understanding intestinal immune modulatory mechanisms by highly virulent *C. perfringens*, which are poorly understood, is critical for the development of new preventive strategies to curb NE (8).

The chicken gastrointestinal tract (GIT) is a key mucosal barrier made up of defined anatomical segments. These segments consist of strategically dispersed and positioned GALT (9, 10) that express pattern recognition receptors (PRR) such as Toll-like receptors (TLR). TLR recognition of microbe-associated molecular patterns (MAMPs) shapes both the microbiome composition (11) and local immune responses to commensal and invading pathogens (12, 13). Exposure to MAMPs (11) leads to DC and macrophage maturation, activation, and subsequent migration to specific areas of the lamina propria (LP) where they modulate immune responses mounted by GALT cells. *C. perfringens* has been shown to alter chicken macrophage function by engaging TLR4 signaling thereby reducing its antibacterial activity (14). However, directly crosslinking TLR1.2, TLR2.1, and TLR15 can improve intestinal responses (15) as well as *in vivo* macrophage antibacterial function against *C. perfringens* (14, 16). Activated macrophages and DCs provide the necessary costimulation for mucosal T-cell maturation (17, 18). Activated mucosal T cells produce either transforming growth factor (TGF)-β or interferon gamma (IFN-γ), key mucosal cytokines that regulate effector functions of macrophage and CD8+ T cells (19). Mature chicken intestinal T cells can directly respond to luminal-derived MAMPs or TLR ligands (20–22). Therefore, the dynamic interaction between macrophages and T cells is important in sustaining the delicate balance between immune activation and regulation (23).

Understanding the role of macrophages and T cells against *C. perfringens* is crucial to establish how the host responds to this bacterium. Competitive interactions in a TLR2-dependent manner are suggested to be effective at negating *C. perfringens*-mediated inhibition of intestinal antibacterial responses. *L. lactis* NZ9000 strain, a Gram-positive food-safe bacterium, possesses a surface capsule rich in LTA motifs (TLR2 ligand) and is free of pathogenic genes (24). Although *L. lactis* is not commonly found in chickens, oral stimulation has been shown to elicit chicken IFN-γ expression *in vivo* suggesting a direct effect on macrophages or T cells, a T helper 1 (Th1) response (25, 26). In contrast, infection with *C. perfringens* leads to an inflammatory process that is directed by a Th2 response (27). Therefore, the aim of the study was to elucidate the potential benefit of *L. lactis* against *C. perfringens* and to elucidate the underlying immunological mechanisms of *L. lactis* in the chicken intestine. An *ex vivo* chicken intestinal mononuclear cell stimulation assay was utilized to define *L. lactis* immune modulatory function in comparison or combination with *C. perfringens*. To that end, chicken mononuclear cell responses such as nitric oxide and IFN-γ production from the various segments of the small intestine suggest a potential role for *L. lactis* to interfere with *C. perfringens* toxin-mediated immune suppression.

## Materials and Methods

### Experimental Animals

Day-old-specific pathogen-free mixed sex white leghorn layers, purchased from the Canadian Food Inspection Agency (CFIA; Ottawa, Canada), were grouped housed in the same isolation units throughout the experiment in specific pathogen-free filtered-air positive pressure rooms. Group housed chickens (*n* = 20) had *ad libitum* access to water and commercial feed. All animal works were approved and performed according to the University of Guelph animal care and use committee guidelines.

### Reagents and Antibodies

The antibodies mouse anti-chicken KULO1-FITC, mouse anti-chicken MHC II-PE, mouse anti-chicken CD3_ζ_-PB, mouse anti-chicken CD4-PE-CY7, mouse anti-chicken CD8α-FITC, and mouse anti-chicken γδTCR-PE were purchased from Southern biotech, Canada. The following mouse anti-chicken CD25-APC (Biorad, Canada) and mouse anti-chicken IFN-γ biotin and streptavidin APC were purchased from Life Technologies, Canada.

#### Reagents

Lipopolysaccharide (stock concentration; 1 mg/ml), phorbol 12-myristate 13-acetate (PMA stock concentration; 100 μg/ml) and ionomycin (ION stock concentration; 1 mg/ml) were all resuspended and stored in DMSO.

### Intestinal Tissue Mononuclear Cell Preparation

Five-centimeter segments of the medial duodenum, jejunum, ileum, and whole cecal tonsil were harvested from 3-week-old white leghorn layers and stored on ice in PBS-containing penicillin (10 U/ml) and streptomycin (10 μg/ml). Each tissue samples were cut into 1-cm segments and washed three times with PBS-containing penicillin (10 U/ml) and streptomycin (10 μg/ml). Tissue samples were subsequently digested with collagenase type 1 (800 U/ml; Millipore-Sigma, ON, Canada) in 4 ml of HBSS buffer (37°C for 20 min) containing penicillin (10 U/ml) and streptomycin (10 μg/ml). Whole tissue digests were applied onto 40-μm BD cell strainers (BD Biosciences, ON, Canada) and crushed through using the flat end of a 10-ml syringe plunger. Duodenum, jejunum, ileum, and cecal tonsil cell suspension were prepared by layering (2:1) onto Histopaque 1077 (Millipore-Sigma, Canada) density-gradient centrifugation and centrifuged at 2,100 rpm (600 × G) for 20 min to allow the separation of mononuclear cells (28). Buffy coat was subsequently aspirated from the interface and washed at 1,500 rpm (400 × G) for 5 min in RPMI-1640 with penicillin (10 U/ml) and streptomycin (10 μg/ml). Mononuclear cells were suspended in complete RPMI cell culture medium; RPMI-1640 medium containing 10% fetal bovine serum (Millipore-Sigma, Canada), penicillin (10 U/ml), and streptomycin (10 μg/ml). Cell number and viability were calculated using a hemocytometer and trypan blue exclusion method. Mononuclear cells were suspended in complete RPMI cell culture medium at a density of 5 × 10^6^ cells/ml and kept on ice. For all assays, mononuclear cells were seeded at a density of 0.5 × 10^6^ cells/200 μl RPMI complete medium in 96 well u-bottom plates.

### Bacterial Strains and Culture Conditions

*Lactococcus lactis* subsp. cremoris strain (nisin^−/−^, NZ9000 strain from MoBiTec GmbH, Göttingen, Germany) was cultured in M17 broth (Gibco, Burlington, ON, Canada) and maintained under anaerobic conditions (30°C and no shaking). The avian highly virulent *C. perfringens* (CP4 isolate) strain was cultured in Brain Heart infusion (BHI) broth (Gibco, Canada) and maintained under anaerobic conditions (37.5°C and no shaking). Overnight cell cultures (OD = 1.215) were washed (4,000 rpm for 10 min) twice and resuspended in PBS. Bacterial cells prepared in PBS were enumerated using a spectrophotometer and subsequently titrated by 10-fold serial dilutions on tryptose sulfite cycloserine (TSC) agar (anaerobic conditions 37.5°C and no shaking). Bacterial titer in accordance with OD reading was used to estimate multiplicity of infection. All bacterial samples were stored until required for specific treatments.

For stimulation studies, optimized multiplicity of infection (MOI) was determined by titrating both *C. perfringens* and *L. lactis* (MOI = 0.001, 0.01, 0.1, and 1.0) on intestinal mononuclear cells to determine an effective treatment concentration that induced a combination nitric oxide (NO) and IFN-γ production with an effect on cell death. MOI of 1 was considered optimized for these specific assays.

### Nitric Oxide Production Using Griess Assay

Duodenum, jejunum, ileum, and cecal tonsil mononuclear cells were seeded in triplicates at a density of 5.0 × 10^5^ cells per well. Mononuclear cells were cultured with medium alone (vehicle), lipopolysaccharide (LPS: 1 μg/ml, positive control; Millipore Sigma, Canada), *C. perfringens* (1 multiplicity of infection; MOI), *L. lactis* (1 MOI), and combination of *C. perfringens* (1 MOI) and *L. lactis* (1 MOI) and incubated (41°C and 5% CO_2_) for 6 and 18 h. Supernatants were collected, and NO production was measured by Griess assay (Promega, Madison, WI, USA), according to the manufacturer’s protocol.

### Flow Cytometry

#### *Ex Vivo* Stimulation Assay

Duodenum, jejunum, ileum, and cecal tonsil mononuclear cells were seeded in triplicates at the density of 5.0 × 10^5^ cells per well. Mononuclear cells were cultured with medium alone (vehicle), LPS (1 μg/ml, positive control; Millipore Sigma, Canada), or PMA (50 ng/ml) plus ION (1 μg/ml), *C. perfringens* (1 MOI), *L. lactis* (1 MOI), and combination of *C. perfringens* (1 MOI) and *L. lactis* (1 MOI) and incubated (41°C and 5% CO_2_) for 18 h. All medium contained Golgi plug and Golgi stop to facilitate intracellular cytokine staining.

#### Apoptosis Assay

To determine the effects of *C. perfringens* on cell apoptosis, mononuclear cells were stained after 18 h with Annexin V-FITC (BD Pharmingen, Mississauga, ON, Canada) and 7-AAD (ThermoFisher Scientific, Mississauga, ON, Canada) in Annexin V staining buffer. Apoptotic and dead cells were acquired on a BD FACS Canto II, and data were analyzed using FloJo software.

#### Intracellular IFN-γ

Eighteen hours post-treatment, mononuclear cells were stained with 7-AAD and subsequently fixed for 30 min using the fixation/permeabilization kit (BD Bioscience, Mississauga, ON, Canada). Cells were incubated in fixation and permeabilization buffer for 30 min at 4°C, blocked (20 min at 4°C) with 1% bovine serum albumin (BSA) followed by incubation (20 min at 4°C) with anti-cIFN-γ-biotin (ThermoFisher Scientific, Canada). Cells were washed twice in staining buffer (1% BSA) and stained with streptavidin-APC (ThermoFisher Scientific, Canada) for 20 min at 4°C in staining buffer. Mononuclear cells were washed twice in staining buffer and incubated with a mouse anti-chicken CD3ζ-PB, mouse anti-chicken CD4-PE-CY7 or mouse anti-chicken CD25-PE-CY7, mouse anti-chicken CD8α-FITC, and mouse anti-chicken γδTCR-PE (Southern Biotech, Canada). All cells were acquired on a BD FACS Canto II, and the data were processed by FlowJo V10 software.

### Statistical Analysis

Graph Pad Prism 8 for windows was utilized to generate graphs and perform statistical analysis. All data are presented as mean + SD and analyzed by unpaired *t*-test. Results were considered statistically significant at ^*^ *p* < 0.05.

## Results

### *Ex Vivo* Treatment With *L. lactis* Improves Responsiveness of Macrophages

**Figure 1:**
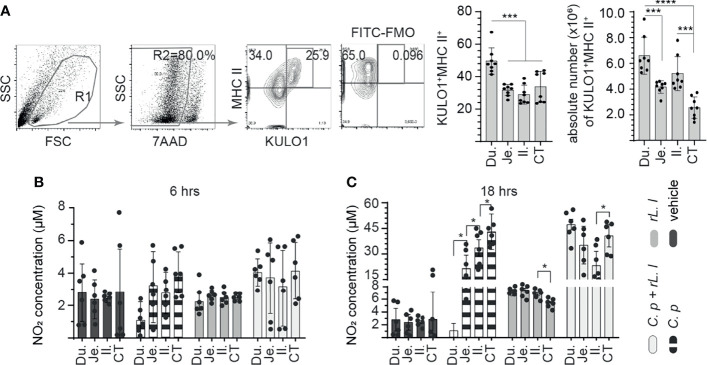
*Ex vivo* induction of NO production by *C perfringens* treatment of intestinal monocyte/macrophages. The frequency of small intestine mononuclear cells isolated from 3-week-old layer chickens (*n* = 8) were analyzed using flow cytometry. **(A)** Dot plots demonstrating the gating strategy utilized to define the frequency and absolute numbers of monocyte/macrophages (KULO1+MHC II+) within the duodenum, jejunum, ileum, and cecal tonsils. The respective small intestine mononuclear cells were stimulated with medium alone (vehicle), LPS (1 μg/ml), *C perfringens* (1 MOI), *L. lactis* (MOI = 1), and combination of *C perfringens* (MOI = 1) and *L. lactis* (MOI = 1). Supernatants were collected at **(B)** 6 and **(C)** 18 h poststimulation and NO concentration were quantified. Paired *t*-test was used to assess normal distribution and test significance. *p < 0.05, ***p < 0.0005 and ****p < 0.0001 indicates a statistically significant difference. The mean ± SD value are shown from three independent experiments performed in triplicates. Du, duodenum; Je, jejunum; Il, ileum; CT, cecal tonsil.

Chicken small intestine macrophage (KULO1+MHC II+) frequency was analyzed in the duodenum, jejunum, ileum, and whole cecal tonsils of 3-week-old layer chickens. A representative FACS dot plot and gating strategy, from cecal tonsil samples, to define the frequency of macrophages in the duodenum, jejunum, ileum, and cecal tonsils is shown (**Figure 1A**). The results demonstrated that the duodenum contains significantly (*p* < 0.0005) more macrophages compared with the jejunum, ileum, and cecal tonsil (**Figure 1A**). No differences were observed in the frequency of macrophages between the jejunum, ileum, and cecal tonsils. The absolute numbers of macrophages within the intestinal segments were also quantified (**Figure 1A**). The results demonstrated significantly (*p* < 0.0005) higher numbers of macrophages within the duodenum when compared with the jejunum and cecal tonsils (**Figure 1A**). No differences were observed in the frequency of macrophages between the duodenum and ileum. The ileum contained significantly (*p* < 0.001) higher numbers of macrophages when compared with cecal tonsils (**Figure 1A**).

Next, intestinal mononuclear cells were stimulated to evaluate their response to *C. perfringens* and *L. lactis* at 6 h (**Figure 1B**) and 18 h (**Figure 1C**) post-stimulation (hps). The results demonstrated no significant difference in NO production at 6 hps in all treatment groups compared with unstimulated control (**Figure 1B**). However, at 18 hps, NO production was significantly (*p* < 0.005) increased from small intestinal mononuclear cells stimulated with *C. perfringens* only or *L. lactis* only compared with unstimulated control (**Figure 1C**). There was a lack of NO production from duodenum mononuclear cells treated with *C. perfringens*. Jejunum mononuclear cells became activated following *C. perfringens* treatment based on NO production when compared with duodenum (*p* < 0.05). Treatment with *L. lactis* enabled the activation of duodenum mononuclear cells in the presence of *C. perfringens* leading to similar NO production when compared with *L. lactis*-treated cecal tonsil mononuclear cells (**Figure 1C**).

### Identification of CD25+ Mononuclear Cells in Various Segments of the Chicken Small Intestine

**Figure 2:**
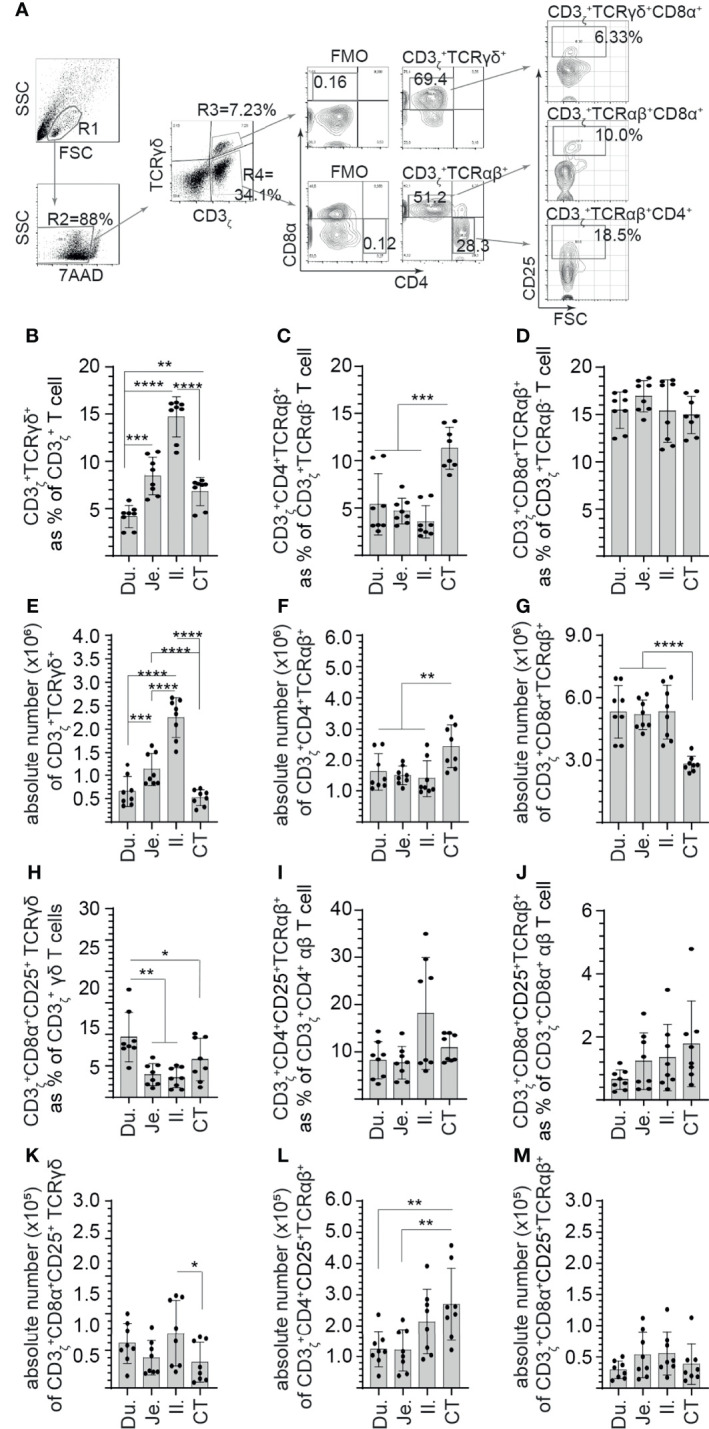
Site-specific differences in the frequency of CD3+ and CD25+ mononuclear cells along the avian small intestine. Small intestine mononuclear cells were isolated from the duodenum, jejunum, ileum, and cecal tonsils of 3-week-old layer chickens (*n* = 8). Live cells were defined based on 7-aminoactinomycin D staining (7AAD-). **(A)** Dot plots demonstrating the gating strategy utilized to define the frequency of **(B)** CD3ζ+CD8α+ γδ T cells, **(C)** CD3ζ+CD4+ αβ T cells, **(D)** CD3ζ+CD8α+ αβ T cells **(H)** CD3ζ+CD8α+CD25+ γδ T cells **(I)** CD3ζ+CD4+CD25+ αβ T cells, and **(J)** CD3ζ+CD8α+CD25+ αβ T cells in the duodenum, jejunum, ileum, and cecal tonsil. Absolute numbers of **(E)** CD3ζ+CD8α+ γδ T cells, **(F)** CD3ζ+CD4+ αβ T cells, **(G)** CD3ζ+CD8α+ αβ T cells **(K)** CD3ζ+CD8α+CD25+ γδ T cells **(L)** CD3ζ+CD4+CD25+ αβ T cells, and **(M)** CD3ζ+CD8α+CD25+ αβ T cells in the duodenum, jejunum, ileum, and cecal tonsil. Paired *t*-test was used to assess normal distribution and test significance. ^*^ *p* < 0.05, ^**^ *p* < 0.001, ^***^ *p* < 0.0005, and ^****^ *p* < 0. 0001 indicated a statistically significant difference. NS indicates no significant difference. The mean ± SD value are shown from six individual layer chickens at 3 weeks of age with staining performed in triplicates Du, duodenum; Je, jejunum; Il, ileum; CT, cecal tonsil.

Next, steady-state and site-specific frequency of CD25+ mononuclear cells within CD3_ζ_+CD4+/CD8α+ αβ and CD3_ζ_+CD8α+ γδ T cells was evaluated. A representative FACS dot plot and gating strategy, from cecal tonsil samples, to define the frequency of CD25+ mononuclear cells in duodenum, jejunum, ileum, and cecal tonsils is shown (**Figure 2A**). The method of tissue digestion and lymphocyte isolation likely leads to isolation of both intraepithelial and lamina propria T lymphocytes. Presence of CD3_ζ_ was used to phenotypically define T cells (CD3_ζ_+CD4+/CD8α+ αβ and CD3_ζ_+CD8α+ γδ T cells). Within the CD3_ζ_+ cells that are TCR γδ+, our results demonstrated that the highest frequency and absolute number of γδ T cells was in the ileum (**Figures 2B, E** **)**. The duodenum had the lowest frequency and absolute number of γδ T cells when compared with the jejunum (*p* < 0.005), ileum (*p* < 0.0001), and cecal tonsil (*p* < 0.05) (**Figures 2B, E** **)**. Negative gating strategy for CD3_ζ_+TCRγδ-T cell was used to define CD3_ζ_+TCRαβ+ T cell that were either CD4+ or CD8α+. CD8α+ αβ T cells were the main αβ T-cell subset found along the avian GIT with the highest absolute numbers (*p* < 0.0001) observed between the duodenum, jejunum, and ileum to that of cecal tonsils (**Figure 2G**). There was no difference in the frequency of CD8α+ αβ T cells between each intestinal segment (**Figure 2D**). However, CD4+ αβ T cells were less abundant than CD8α+ αβ T cells. The frequency and absolute number of CD4+ αβ T cells was significantly higher (*p* < 0.001) in the cecal tonsils compared with the duodenum, jejunum, and ileum (**Figures 2C, F** **)**. To further differentiate the identified T-cell subsets, CD25 was used to define specific subsets within CD3_ζ_+CD4+/CD8α+ αβ and CD3_ζ_+CD8α+ γδ T cells. The results demonstrated with regard to CD3_ζ_+ T cells, CD25+ γδ T cells were significantly (*p* < 0.01) more abundant in the duodenum compared with the jejunum, ileum, and cecal tonsil (**Figure 2H**). In comparison with the cecal tonsils, significantly lower (*p* < 0.05) absolute number of CD25+ γδ T cells was detected (**Figure 2K**). However, this subset represented a minor population of γδ T cells that reside in the intestine (<0.8% in γδ T). In αβ T cells, the results presented here demonstrated no significant difference in the frequency of CD4+CD25+ αβ T cells (**Figure 2I**) or CD8α+CD25+ αβ T cells (**Figure 2J**) between the duodenum, jejunum, ileum, and cecal tonsil. Based on absolute numbers, CD4+CD25+ αβ T cells were more abundant (*p* < 0.01) in the cecal tonsil compared with the duodenum and jejunum (**Figure 2L**). CD8α+CD25+ αβ T cells were incrementally higher from the proximal (duodenum) to distal small intestine (cecal tonsil), but no significant differences were observed between the specific intestinal sites (**Figures 2J, M** **)**. In addition, CD4+CD25+ αβ T cells were significantly more abundant (*p* < 0.01) based on frequency compared with CD8α+CD25+ αβ T cells.

### *Ex Vivo* Stimulation With *L. lactis* and *C. perfringens* Differentially Modulates the Frequency of CD25+ Small Intestine Mononuclear Cells

**Figure 3:**
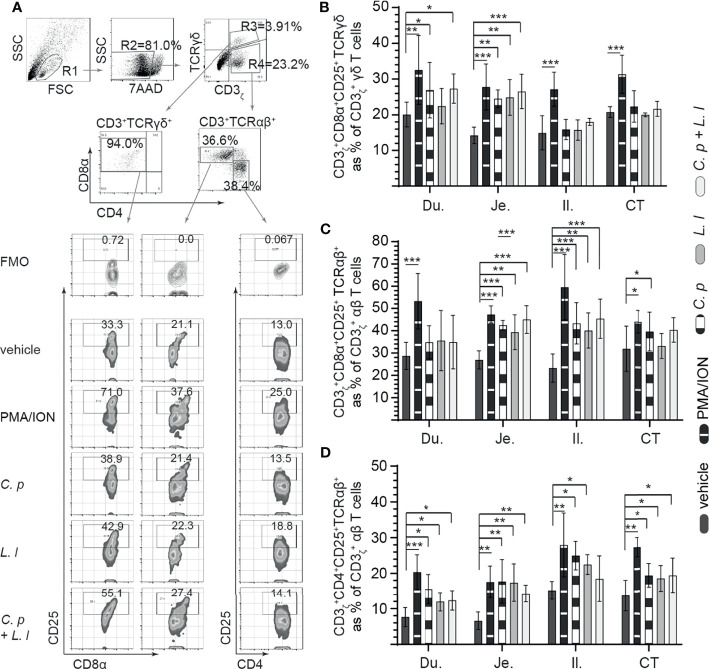
*Ex vivo* induction of CD25+ small intestinal mononuclear cells by both *C perfringens* and *L. lactis*. Small intestine mononuclear cells (*n* = 6) were isolated from the duodenum, jejunum, ileum, and cecal tonsils. Cells were stimulated with medium alone (vehicle), PMA (50 ng/ml) plus ION (1 μg/ml), *C perfringens* (1 MOI), *L. lactis* (MOI = 1), and combination of *C perfringens* (MOI = 1) and *L. lactis* (MOI = 1). Mononuclear cells were analyzed at 18 h poststimulation by flow cytometry for surface expression of CD25. **(A)** Dot plots and density plot demonstrating the gating strategy utilized to define the frequency of **(B)** CD3ζ+CD8α+CD25+ γδ T cells, **(C)** CD3ζ+CD8α+CD25+ αβ T cells, and **(D)** CD3ζ+CD4+CD25+ αβ T cells from the duodenum, jejunum, ileum, and cecal tonsil. Paired *t*-test was used to assess normal distribution and test significance. *(p < 0.05), **(p < 0.001) and ***(p < 0.0005) indicates a statistically significant difference. The mean ± SD value are shown from three independent experiments performed in triplicates. Du, duodenum; Je, jejunum; Il, ileum; CT, cecal tonsil.

Small intestinal mononuclear cells were stimulated *ex vivo* with *L. lactis* (MOI = 1) only, *C. perfringens* (MOI = 1) only, or *L. lactis* (MOI = 1) in combination with *C. perfringens* (MOI = 1). Cell surface expression of CD25 was assessed at 18 hps (**Figure 3**). PMA and ION were utilized as positive control and medium alone as vehicle (unstimulated). A representative FACS dot plot and gating strategy, from ileum samples, to define CD25+ cells poststimulation in the duodenum, jejunum, ileum, and cecal tonsils is shown (**Figure 3A**). The results demonstrated that stimulation with PMA and ion led to a significant increase (*p* < 0.001) in cell surface expression of CD25 in γδ T cells (**Figure 3B**), CD8+ αβ (**Figure 3C**), and CD4+ αβ (**Figure 3D**) T cells when compared with vehicle-stimulated cells. Duodenum and jejunum γδ T cells compared with ileum and cecal tonsil cells were more responsive to *ex vivo* stimulation with *C. perfringens* leading to a signification increase (*p* < 0.05) in cell surface expression of CD25 in γδ T when compared with vehicle-treated cells (**Figure 3B**). Both ileum and cecal tonsil γδ T cells demonstrated a lack of responsiveness to *ex vivo* stimulation with both *L. lactis* and *C. perfringens* when compared with vehicle-treated cells (**Figure 3B**).

With respect to CD8α+ αβ T cells, *ex vivo* stimulation with *L. lactis* or *C. perfringens* alone and *L. lactis* in combination with *C. perfringens* induced a significant increase (*p* < 0.05) in cell surface expression of CD25 in jejunum and ileum CD8α+ αβ T cells when compared with vehicle-treated cells (**Figure 3C**). However, *ex vivo* stimulation with *L. lactis*, *C. perfringens*, and *L. lactis* in combination with *C. perfringens* had no effect on cell surface expression of CD25 in duodenum and cecal tonsil CD8α+ αβ T cells when compared with vehicle-treated cells (**Figure 3C**). In CD4+ αβ T cells, the results demonstrated a significant increase (*p* < 0.05) in cell surface expression of CD25 in CD4+ αβ T cells stimulated *ex vivo* with *L. lactis* or *C. perfringens* alone when compared with vehicle in all sites of the small intestine (**Figure 3D**).

### *Ex Vivo* Stimulation With *L. lactis* Elicits Small Intestinal Mononuclear Cells IFN-γ Production

**Figure 4:**
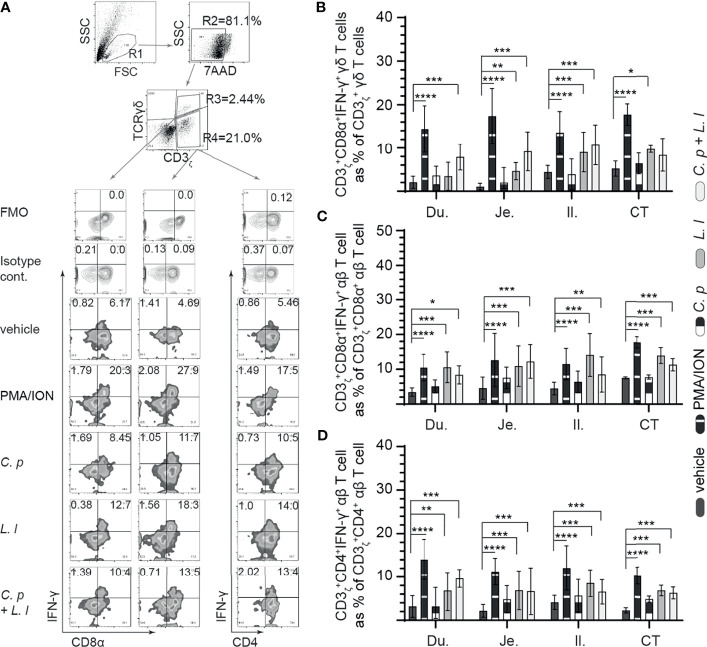
*Ex vivo L. lactis* treatment elicited intracellular IFNγ expression in intestinal mononuclear cells. Small intestine mononuclear cells (*n* = 6) were isolated from the duodenum, jejunum, ileum, and cecal tonsils. Cells were stimulated with medium alone (vehicle), PMA (50 ng/ml) plus ION (1 μg/ml), *C perfringens* (1 MOI), *L. lactis* (MOI = 1), and combination of *C perfringens* (MOI = 1) and *L. lactis* (MOI = 1). Cells were harvested at 18 h poststimulation, and data were analyzed for frequency of IFNγ+ cells using flow cytometry. **(A)** Dot plots and density plot demonstrating the gating strategy utilized to define the frequency of **(B)** CD3ζ+CD8α+IFNγ+ γδ T cells, **(C)** CD3ζ+CD8α+IFNγ+ αβ T cells, and **(D)** CD3ζ+CD4+IFNγ+ αβ T cells. Paired *t*-test was used to assess normal distribution and test significance. ^*^ *p* < 0.05, ^**^ *p* < 0.001, ^***^ *p* < 0.0005, and ^****^ *p* < 0.0001 indicate a statistically significant difference. The mean ± SD value are shown from three independent experiments performed in triplicates. Du, duodenum; Je, jejunum; Il, ileum; CT, cecal tonsil.

IFN-γ is a key cytokine secreted by activated (αβ and γδ) T cell (29). Small intestinal mononuclear cell activation, based on IFN-γ production, was assessed in response to *ex vivo* stimulation with *L. lactis* (MOI = 1), *C. perfringens* (MOI = 1) only, or *L. lactis* (MOI = 1) in combination with *C. perfringens* (MOI = 1). A representative FACS dot plot and gating strategy, from jejunum samples, to define IFN-γ+ cells in the duodenum, jejunum, ileum, and cecal tonsils is shown (**Figure 4A**). The results demonstrated that stimulation with PMA and ion led to a significant increase (*p* = 0.05) in IFN-γ+ cells among γδ T cells (**Figure 4B**), CD8+ αβ T cells (**Figure 4C**), and CD4+ αβ (**Figure 4D**) T cells when compared with vehicle-stimulated cells. T cells were observed to be more hyporesponsive to *C. perfringens* treatment in contrast to *L. lactis* based on the frequency of IFN-γ+ cells. Analysis for frequency of IFN-γ+ cells indicate that *L. lactis*, in contrast to *C. perfringens*, is a strong inducer (*p* < 0.01) of γδ (**Figure 4B**) and αβ (**Figures 4C, D** **)** T-cell ability to produce IFN-γ. The results demonstrated that *ex vivo* stimulation with *C. perfringens* did not result in cellular activation based on no changes in the frequency of IFN-γ+ γδ T cells (**Figure 4B**) and IFN-γ+ αβ T cells (**Figures 4C, D** **)** when compared with vehicle-treated cells in the duodenum, jejunum, ileum, and cecal tonsil.

In γδ T cells, there was a significant increase (*p* < 0.01 and *p* < 0.001) in the frequency of IFN-γ+ γδ T cells from the jejunum, ileum, and cecal tonsil-stimulated *ex vivo* with *L. lactis* when compared with *C. perfringens* or vehicle-treated cells (**Figure 4B**). However, *ex vivo* stimulation with *C. perfringens* had no effects on the frequency of small intestinal IFN-γ+ γδ T cells. The combination of *ex vivo* stimulation with *L. lactis* and *C. perfringens* together elicited γδ T-cell activation, particularly in the duodenum due to the significant increase (*p* < 0.01) in IFN-γ+ γδ T cells when compared with vehicle-treated cells (**Figure 4B**). αβ T cells from the duodenum, jejunum, ileum, and cecal tonsil were responsive to *ex vivo* stimulation with *L. lactis* based on the significant increase (*p* < 0.01) in the frequency of CD8α+IFN-γ+ αβ T (**Figure 4C**) and CD4+IFN-γ+ αβ T (**Figure 4D**) cells compared with vehicle-stimulated cells. In addition, the results demonstrated that *ex vivo* stimulation with *L. lactis* in combination with *C. perfringens* resulted in a significant increase (*p* < 0.01) in the frequency of both CD8α+IFN-γ+ αβ T (**Figure 4C**) and CD4+IFN-γ+ αβ T (**Figure 4D**) cells compared with vehicle- or *C. perfringens*-treated cells. Differential responses were observed in *C. perfringens*-stimulated αβ T cells (**Figures 4C, D** **)**. The results demonstrated that e*x vivo* stimulation with *C. perfringens* resulted in a significant increase (*p* < 0.01) in jejunum and cecal tonsil CD8α+IFN-γ+ αβ T cells (**Figure 4C**) and jejunum, ileum, and cecal tonsil CD4+IFN-γ+ αβ T cells (**Figure 4D**) when compared with vehicle-treated cells.

### *Ex Vivo L. lactis* Stimulation Limits *C. perfringens*-Induced Apoptosis of Small Intestinal Mononuclear Cells

**Figure 5:**
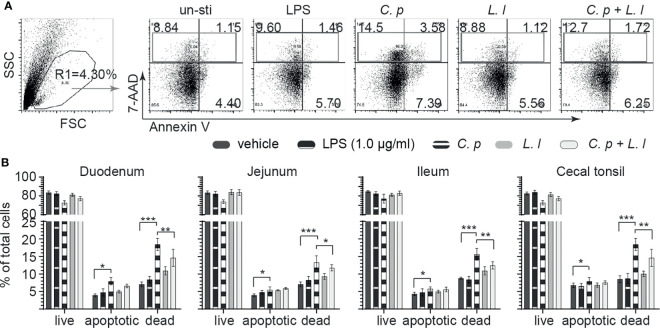
*Ex vivo L. lactis* treatment improves small intestinal mononuclear cells resistance to *C perfringens-*induced apoptosis. Small intestine mononuclear cells (*n* = 6) isolated from the duodenum, jejunum, ileum, and cecal tonsils were stimulated with medium alone (vehicle), LPS (1 μg/ml), *C perfringens* (1 MOI), *L. lactis* (MOI = 1), and combination of *C perfringens* (MOI = 1) and *L. lactis* (MOI = 1). Gut mononuclear cells harvested at 18 h poststimulation were stained with 7AAD (dead cell marker) and Annexin V (early apoptotic marker). **(A)** Dot plots demonstrating the gating strategy utilized to define live (7AAD-Annexin V-), dead (7AAD+Annexin V+), and apoptotic (7AAD-Annexin V+) mononuclear cells from the **(B)** duodenum, jejunum, ileum, and cecal tonsil. Paired *t*-test was used to assess normal distribution and test significance. ^*^ *p* < 0.05, ^**^ *p* < 0.001, and ^***^ *p* < 0.0005 indicate a statistically significant difference. The mean ± SD value are shown from three independent experiments performed in triplicates.

*C. perfringens* is well documented to express an array of toxins that can modulate both cellular activation and induce necrotic and apoptotic signaling in epithelial cells (30). Small intestinal mononuclear cells isolated from the duodenum, jejunum, ileum, and cecal tonsil were stained with 7AAD and Annexin V at 18 h post-*ex vivo* stimulation with *C. perfringens* (MOI = 1), *L. lactis* (MOI = 1), or a combination of *C. perfringens* (MOI = 1) and *L. lactis* (MOI = 1) or vehicle (PBS) only. LPS (1 μg/ml) was used as reaction control. The percentages of live cells (7AAD^−^Annexin V^−^), apoptotic cells (7AAD^−^Annexin V^+^) and dead cells (7AAD^+^Annexin V^−^ + 7AAD^+^Annexin V^+^) were determined using flow cytometry. A representative FACS dot plot and gating strategy, from cecal tonsil (CT) samples, is shown demonstrating gating strategy for duodenum, jejunum, ileum, and cecal tonsil mononuclear cells (**Figure 5A**). The results demonstrated that *ex vivo* stimulation with *C. perfringens* led to a significant increase (*p* = 0.0001) in both dead and apoptotic mononuclear cells as isolated from the duodenum, jejunum, ileum, and cecal tonsil when compared with vehicle- or LPS-treated cells (**Figure 5B**). However, *ex vivo* stimulation with *L. lactis* in combination with *C. perfringens* resulted in a significantly lower (*p* < 0.01) frequency of both dead and apoptotic mononuclear cells from the duodenum, jejunum, ileum, and cecal tonsil when compared with *C. perfringens* only treated cells (**Figure 5B**). *Ex vivo* treatment with *L. lactis* tended to increase both the frequency of dead and apoptotic mononuclear cells from the duodenum, jejunum, ileum, and cecal tonsil but was not significant when compared with vehicle- or LPS-treated cells (**Figure 5B**).

## Discussion

In the food technology sector, *L. lactis* has been successfully applied to limit *Listeria monocytogenes* (31) and *Staphylococcus aureus* (32). Due to its designated food-safe status, *L. lactis* has been applied in various animal models (32–34), including in chickens, as an oral vaccine vector against avian influenza virus (AIV), Newcastle disease virus (NDV), and infectious bronchitis virus (IBV) (24, 35, 36). *L. lactis* has received little attention with respect to its potential intestinal modulatory activities in poultry production systems (24) as it is not considered to be commensal (37). Clear direct evidence in a mouse inflammatory model indicates that *L. lactis* NZ9000 can reverse intestinal inflammatory processes (38). Considering that *C. perfringens* primary infection as an opportunistic bacteria requires intestinal inflammation and injury for progression of NE, application of beneficial bacteria such as *L. lactis* with anti-inflammatory activity (38) in chickens can provide insights for induction of a protective innate and adaptive immunes response against *C. perfringens* (15).

Distinct pathogenic avian *C. perfringens* strains have the ability to produce a wide array of toxins such as α-toxin, NetB, and TpeL, which constitute the underlying mechanisms for lesions associated with NE (6, 38). Loss of intestinal epithelium in cases of avian NE is a direct consequence of disrupting the lamina propria cellular activities, extracellular matrix, and epithelial intercellular junctions (39). The highly virulent *C. perfringens* strain utilized in this experiment actively secretes NetB, TpeL, and α-toxin with known function in disrupting the GALT and intestinal physical barrier (40). In a mouse infection model, α-toxin has been shown to effectively modulate macrophage activation (41) with limited impact on its replication (42). In chickens, NetB and TpeL affect cellular morphology leading to pore formation resulting in cell death (4, 30, 43). No NE is observed in chickens infected with *C. perfringens* strain lacking NetB and TpeL (30). Therefore, a direct relationship exists between NetB, TpeL, and progression of NE. Our results provide further insight into this relationship. In fact, *ex vivo* treatment of intestinal mononuclear cells with *C. perfringens* did lead to significantly more mononuclear cell death than untreated cells or *L. lactis* only treated cells. NetB, TpeL, and α-toxin may be able to directly modulate immune system cells. Previous studies have demonstrated that probiotics are able to modify their microenvironment which is not conducive for growth of potentially competing bacteria. *L. lactis* is well known to produce antimicrobial substances such as lactic acid and acetic acid (24) in addition to nisin. The *L. lactis* cremoris used in this study is a nisin knockout therefore elucidating to other mechanism of antimicrobial effects. These antimicrobial substances can potentially affect the pH of the culture system and decrease toxin function. Similarly, in chickens, *lactobacillus* probiotic bacteria have been demonstrated to change the pH of the milieu that led to a decreased in *C. perfringens* proliferation and toxin production (8). In accordance with previous observations, the fact that treatment together with *L. lactis* could limit *C. perfringens*-induced cell death demonstrates a potential role in either limiting toxin production or their functional activity. Current work in our lab is attempting to elucidate further *L. lactis* cremoris antimicrobial mechanisms against avian pathogens such as *C. perfringens*.

Although not yet fully elucidated, it is likely that avian macrophages are highly susceptible to *C. perfringens*-derived toxins including α-toxin. In fact, our results demonstrate that macrophages make up more than 40% of all immune system cells that populate the chicken intestine. The duodenum was more populated by macrophages in comparison with the jejunum, ileum, and cecal tonsil. The avian *C. perfringens* has been shown to alter a chicken macrophage cell line function by engaging TLR4 signaling thereby reducing its antibacterial activity and increasing inflammatory processes (14). It is well established that human T cells in the presence of M1 macrophages or DC secrete a large amount of IFN-γ with little IL-4, IL-5, and IL-10 (44). However, this mechanism is unclear in the chicken mucosal immune system (45). This study also provides further evidence that *ex vivo* treatment with *C. perfringens* could effectively limit primary intestinal macrophage activation based on NO production even after 18 h of culture. This is supported by the fact that stimulation with *L. lactis* on its own or in the presence of *C. perfringens* increased intestinal macrophage activation. *L. lactis* can be immunogenic whereas macrophages might be unresponsive to presence of *C. perfringens* when compared with *L. lactis*. Lack of cellular activation can be either due to an increase in *C. perfringens*-induced cell death or cellular unresponsiveness. The possibility based on our observation and that of others that *C. perfringens* immune modulation by suppressing or redirecting innate cell activation cannot be excluded (46). Innate cell activation is crucial for adaptive immune system cells such as T cell.

In this study, the results presented demonstrate that a substantial portion of intestinal T cells expressed CD25. The interleukin-2 receptor alpha chain (also called CD25) is considered a determinant of T-cell functional ability. Several cytokines secreted by monocytes and macrophages trigger T-cell activation and induction of CD25 expression. CD25 defines two major T-cell subsets, effector T cells based on IFN-γ expression and regulatory T cells (T_reg_) that are expressing TGF-β (47, 48). One of the limitations of avian research is diversity of antibodies for extensive phenotypic analysis of avian T cells (28). As such, dual staining of IFN-γ and TGF-β is a limiting part of defining and differentiating CD25+ T-cell subsets. In this study, CD25+ T cells define the intestinal mononuclear cells with potential for either an effector or regulatory role. Mature, differentiated T cell express TLRs and can directly respond to MAMPS. Direct stimulation with TLR agonists can promote more effective T-cell-mediated immunity. In mice, activated T cell, IFN-γ expressing cells, can either support B-cell activation, T-dependent B-cell activation, or enhance the mucosal barrier against invading microbes by supporting innate cells such as macrophages (49). Intestinal IFN-γ is a key mucosal mediator. The IFN-γ signaling pathway coordinates several biological responses, primarily involved in host defense and immune surveillance by enabling “classical” activation of human macrophages (M1) and cytotoxic CD8+ T cells (50). Through TLR-mediated signaling, some probiotics, in chicken, may alter cytokine production by promoting nuclear export of NF-κB or interferon regulatory factor (IRF) 1 which leads to expression of Th2 proinflammatory cytokines such as IL-4 and IL-10 or Th1 cytokines such as IFN-γ, respectively (51), cytokines that work in opposing fashions. *C. perfringens* strains are encapsulated by different combination of atypical LTA motifs thereby altering their immunogenicity. TLR stimulation may be limited to either TLR2 or TLR4 and not TLR21. It is possible, in chickens, that highly virulent *C. perfringens* strains are least immunogenic in terms of innate sensing (52). *In vivo* experiments have also demonstrated that *C. perfringens*-induced TLR signaling in chickens is not sufficient to elicit cytokine expression such as IFN-γ due in part to an increase in IL-4 and IL-10 cytokine expression (14, 22). As demonstrated in this study based on intracellular IFN-γ staining in T cells, the results presented here demonstrated that *ex vivo* stimulation of intestinal mononuclear cells with *C. perfringens* did not elicit T-cell activation. It is likely that *C. perfringens* is a poor immunogen in part due to its cellular structure but also the functional role played by the vast array of toxins (NetB, TpeL, and α-toxin) it produces. Treatment with *L. lactis* on its own elicited cellular activation based on detection of intracellular IFN-γ in γδ T cells and αβ T cells. *L. lactis* is not common to chickens but is immunogenic, demonstrating that TLR crosslinking is effective at eliciting a T-cell response. More importantly, treatment with *L. lactis* in combination with *C. perfringens* led to cellular activation based on detection of intracellular IFN-γ in γδ T cells and αβ T cells when compared with *C. perfringens*-stimulated cells or stimulated cells. Taken together, these results are consistent with observation that an ability for *L. lactis* to limit cell death, in a similar manner as probiotic bacteria, can increase T-cell responsiveness to stimulation, even in the presence of *C. perfringens*.

Expression of CD25 is often associated with regulatory T (Treg) cells that are known to inhibit IFN-γ+ T-cell effector responses. In contrast, IFN-γ+ can activate Treg but limit their ability to secret TGF-β but not IL-10 (53). Here, we demonstrate that a subset of chicken intestinal (duodenum, jejunum, ileum, and cecal tonsil) CD8+ γδ T cells and CD4+ αβ T cells expressed CD25. Recently, chicken cecal tonsils and spleen CD25+ T cell have been shown to have a regulatory function with the ability to express IL-10 and TGF-β thereby limiting effector T-cell functions such as proliferation and cytokine production (47, 48). The results of the present study demonstrated that *ex vivo* treatment of chicken intestinal mononuclear cells with a highly virulent *C. perfringens* bacteria led to an increase in expression of CD25 in T cells but not IFN-γ, an indication for two functionally distinct T-cell subsets. In mice, presence and recognition of colonic *Clostridia* is essential for induction and maintenance of tissue-resident CD25+ cells with regulatory (TGF-β+) function (54). By contrast, in mice, the presence of commensal or nonpathogenic bacterial DNA was shown to limit Treg conversion (55). In chickens, infection with a highly virulent *C. perfringens* led to a decrease in intestinal TGF-β mRNA expression but an increase in IL-10 (27). CD25+ Treg cells can express IL-10 to suppress cellular activation. In contrast, IFN-γ+-expressing T cells can activate CD25+ Tregs in turn limiting their ability to produce IL-10 but increase TGF-β expression which could be critical to limit *C. perfringens*-induced NE. During infection with *C. perfringens*, cell death induced by NetB, TpeL, and α-toxin is a key factor for NE progression. Immune unresponsiveness and a lack of TGF-β expression, essential for tissue remodeling and repair, likely due to induced expression of IL-10 in intestinal epithelial and immune system cells, can exacerbate NE. In addition, our results demonstrate that *L. lactis* treatment also increased the frequency of CD4+CD25+ αβ T cell and CD8+CD25+ γδ T cells *ex vivo*. Therefore, *de novo* induction of CD25+ T cell could be essential for macrophage function and subsequent effector T-cell (IFN-γ+) activation.

Improving intestinal health is the most important issue currently being tackled by the poultry industry. Here, we have shown that *L. lactis* can directly activate mucosal γδ and αβ T cells based on intracellular IFN-γ staining. These effects were associated with limiting *C. perfringens*-induced cell death and promoting macrophage activation. Taken together, this body of work demonstrates the potential feasibility of *L. lactis* application against *C. perfringens* as a modulator of intestinal immune responses in the chicken.

## Data Availability Statement

The raw data supporting the conclusions of this article will be made available by the authors, without undue reservation.

## Ethics Statement

The animal study was reviewed and approved by University of Guelph animal care and use committee.

## Author Contributions

NB and SS designed the experiment. NB performed the experiments, collected and analyzed the data, and wrote the first draft of the manuscript. MA, BS, RK, and SS critically reviewed the manuscript. SS and RK critically reviewed the flow cytometry data. All authors read and approved the final manuscript.

## Conflict of Interest

The authors declare that the research was conducted in the absence of any commercial or financial relationships that could be construed as a potential conflict of interest.

## Publisher’s Note

All claims expressed in this article are solely those of the authors and do not necessarily represent those of their affiliated organizations, or those of the publisher, the editors and the reviewers. Any product that may be evaluated in this article, or claim that may be made by its manufacturer, is not guaranteed or endorsed by the publisher.
